# How are proportionate universalist services affected by resource constraint in practice? Exploring implementation challenges using a synthesis of four health visiting studies in England

**DOI:** 10.1136/jech-2026-225934

**Published:** 2026-07-16

**Authors:** Alison Lamont, Katie Harron, Jane Barlow, Catherine Bunting, Rebecca Cassidy, Amanda Clery, Sally Kendall, Mengyun Liu, Joanna L Lysons, Louise McGrath-Lone, Jenny Woodman

**Affiliations:** 1 Social Research Institute, University College London, London, UK; 2 Great Ormond Street Institute of Child Health, University College London, London, UK; 3 Department of Social Policy and Intervention, University of Oxford, Oxford, UK; 4 Department of Medical Statistics, London School of Hygiene and Tropical Medicine, London, UK; 5 Centre for Health Services Studies, University of Kent, Canterbury, UK; 6 Population, Policy and Practice Department, University College London, London, UK; 7 Centre for Child, Adolescent and Family Research, University of Cambridge, Cambridge, UK

**Keywords:** Health inequalities, HEALTH POLICY, HEALTH SERVICES, NEEDS ASSESSMENT, PUBLIC HEALTH

## Abstract

**Background:**

Proportionate universalism aims to improve overall health while decreasing health inequity across the social spectrum. For health visiting in England, the ‘universal’ offer is five health reviews for children aged under 5 years, with ‘proportionate’ support provided through additional health visiting contacts when greater need is identified.

**Methods:**

We synthesised the published findings of four mixed-method studies into variation in health visiting services in England to examine how proportionate universalism works in practice. The studies analysed Community Services Dataset data from 2016–2020 and interviews and observations with parents and professionals (collected 2023–2024).

**Results:**

Between 2018 and 2020, up to 98% of infants in 57 areas received their universal contacts, even as services experienced reduced funding and staffing, indicating high reach. However, interview data suggest universal reach was widely achieved by offering a differentiated version of universal contacts based on whether families already have known needs or not. This affects health visiting’s ability to identify need across the social spectrum. Similarly, additional contacts were found to be widespread. Health visitors typically reported using these to support families with known complex needs that fell short of thresholds for other services.

**Conclusions:**

In an environment of resource constraint and limited specialist services, a proportionate universalist service will be pushed away from identifying unknown needs and pulled towards supporting the most acute known needs. This may reduce the quality of the universal service, even if reach is maintained. Implications for policymakers and service commissioners are discussed.

**WHAT IS ALREADY KNOWN ON THIS TOPIC**

- Proportionate universalism (Marmot *et al,* 2010) describes a model for the provision of public health services, such as health visiting, whereby service provision should be universal but with an intensity and scale that is proportional to the level of disadvantage. While the conceptual scope of proportionate universalism has been developed (Carey *et al,* 2015; Francis-Oliviero *et al,* 2020), there are currently few studies that discuss its utility in practice, and there are no studies that we know of that systematically use empirical studies to explore how proportionate universalism operates in practice.

**WHAT THIS STUDY ADDS**

- This study suggests that proportionate universalism’s drive to both reduce inequalities and improve health along every point of the social spectrum means that additional needs can always be identified. However, not all needs can be met because resources are limited. Second, in the case of health visiting, the analysis suggests that services tend to manage the amount of need they are likely to uncover by offering different versions of the ‘universal’ service for those who already have known needs, compared with other families.

**HOW THIS STUDY MIGHT AFFECT RESEARCH, PRACTICE OR POLICY**

- These findings offer valuable insights for researchers and policymakers interested in the implementation or design of public and community health services by evidencing how proportionate universalist services must operate within resource constraint, forcing decisions to be made about how to rationalise service spending and demands on the workforce. For health visiting, this manifests in offers of different versions of the ‘universal’ service for families based on whether they have already known needs or not. This pushes the ‘proportionate’ part of the service towards supporting already-known needs, while cost-saving measures are directed at the ‘universal’ part of the service, which reduces the likelihood of eliciting as-yet-unknown needs, impacting the quality of universal needs elicitation. This undermines the preventative potential of proportionate universalist service design and impacts the service’s potential to reduce inequalities across the spectrum.

## Introduction

 Proportionate universalism has attracted significant attention since the Marmot Review (2010)^1^ coined the term. It purports that, to increase health at every point of the spectrum while decreasing health inequalities, ‘actions should be universal, but with an intensity and a scale that is proportional to the level of disadvantage’.^1^ This highlights the need for both universal interventions aimed at improving population health and additional targeted interventions for those parts of the population with greater need.^2^ Proportionate universalism, therefore, articulates a theory of how to organise services according to the public health principle of prevention.

The UK National Health Service (NHS) reflects this approach: ‘universal care, free at the point of delivery, based on need and funded through general taxation’.^3^ Prevention is one of the ‘three shifts’ driving current UK health policy and framed as the most cost-effective way of addressing ill-health.^4^ Similarly, proportionate universalism has attracted international attention^5^ because it promises an equitable means of improving health under conditions of slowing economic growth and increasing welfare expenditure by nation-states.^6^ It is seen as a progressive and redistributive approach to public health in comparison with purely ‘universal’ services, which may be costly or purely ‘targeted’ services, which may be stigmatising.^6^ It is, therefore, important to explore how proportionate universalism performs in practice in increasingly resource-constrained welfare states and healthcare systems, such as England. While Marmot and colleagues^1 7^ and others^8 9^ have developed the theoretical foundation of proportionate universalism, there are currently few studies that explore how the theory has been translated into service organisation and delivery within resource-constrained environments. Current evidence highlights the difficulty in eliciting and responding to beyond-universal need without using stigmatising^10^ or arbitrary thresholds^11^ and the complexity of developing a shared understanding of proportionate universalism across services.^5 12^ To provide analysis which can help inform national and local public health policymakers, commissioners and providers’ decision-making, we explore empirically how the unlimited health ambition of proportionate universalism is squeezed into limited budgets and what happens to those services that exist without formalised thresholds to triage and prioritise cases.

### Health visiting in England as a proportionate universalist service facing resource constraint

The ‘universal’ part of proportionate universalism in health visiting means that any family with a child aged under 5 years should be offered five Health and Development Reviews (hereafter, ‘universal contacts’), delivered by their local health visiting team at set periods of the child’s life: antenatally at 28 weeks; a new birth contact within 14 days of birth; a 6–8-week review; a 12-month review; and a 2–2½-year review(see further, Box 1). Universal contacts have a dual purpose: health promotion and identifying child and family need.^13^

Box 1 **Health visiting in England**

Health visiting is a well-established public health service offered to all families with a child aged under 5 years in England, mandated as part of the Healthy Child Programme. Healthy Child Programme services focus on ‘improving health outcomes and reducing inequalities at individual, family and community levels’^14^ and are designed according to proportionate universalism.^32 33^ Since 2015, health visiting in England has been commissioned by local authorities. Currently, the National Health Service (NHS) is the provider for health visiting services in most local authorities in England, although in some areas, services are provided by local government or by private providers.^31^

Health visitors are specialist public health community nurses with master’s level education in community public health nursing.^13^ Health visitors may have their own named caseload of families, operate under a ‘corporate’ model where families are seen by the first available health visitor or some combination of the two.^34^ Health visitors lead skill-mix teams that typically include community nursery nurses and community staff nurses or their equivalents. These roles are less qualified and lower paid than health visitors’ and should carry less responsibility. Community nursery nurses and equivalents usually have a diploma in childcare and/or child development and are typically employed as Band 3 or 4 on the NHS pay scale. Community staff nurses and their equivalents usually have a degree-level clinical qualification in nursing and are typically employed as Band 5 on the NHS pay scale. Health visitors are usually employed at Band 6 in the NHS pay scale, where a higher band level indicates a higher pay bracket. There is regional variation in how skill mix is used in delivering health visiting and how they are integrated with health visiting teams.^31^

Health visiting works through contacts made with families by a member of the health visiting team. Delivering ‘contacts’ with families can involve making a home visit, making a telephone call or inviting families into a health and social care setting such as a family hub or general practitioner’s surgery. It can involve the delivery of the universal content across several different contacts or in one longer contact arranged specifically for that purpose.^31^ A contact can be delivered by a health visitor or a skill-mix member of the team.

In principle, any caregiver with a child aged under 5 years can have an unlimited number of contacts with their health visiting team about their child’s health-related issues until the child ages out of the service. There are no specific regulations on how these additional contacts are decided or delivered or how many a family may receive. These decisions are made by health visiting teams in response to the needs elicited using professional judgement^35^ and standardised measures, such as the Edinburgh Postnatal Depression Scale.^36^

The ‘proportionate’ increase in scale and intensity of health visiting is provided through additional contacts: ‘additional contacts are suggested for consideration where health visitors, or a member of their team, could respond to a family’s identified needs’.^14^ Where the need elicited is beyond what health visiting can meet, the service signposts to relevant services, such as food banks or support groups, or refers to specialists such as paediatricians, speech and language therapists or children’s social services.^14^

A key driver for regional variation in health visiting is resource constraint.^15^ Health visiting is funded through the public health grant, made by the Department of Health and Social Care to local authorities, and shared with other public health initiatives. The Health Foundation^16^ estimates that there was a 28% real-term, per-person cut in the value of the Public Health Grant between 2015 and 2024. Between 2015 and 2023, the Institute of Health Visiting used published workforce data to show a parallel 40% reduction in full-time equivalent health visitors in England.^17^ In the Institute of Health Visiting’s 2025 annual survey of their health visitor members, 84% of survey respondents reported increased demand for their service in the last 12 months across the UK; and in England, 74% reported that workforce shortages impacted their ability to deliver universal contacts.^17^ The decrease in funding and health visitor numbers has coincided with wider trends which increase health visiting demand. This includes increasing rates of children living in poverty,^18^ childhood obesity^19^ and adult mental health conditions.^20^

The targeted services to which health visitors refer specialist needs are also experiencing resource constraint in a context of rising need. Bilson and Martin found increased numbers of referrals to children’s social services^21^ during in the 2010s, the same period in which Webb found that local authorities had less money to spend on the children they had a legal duty to support.^22^ Likewise, while England has developed perinatal maternal and infant mental health teams to which health visiting can refer, these are under-resourced for the level of demand, leading to waiting lists and patchy provision even when referral is successful.^20 23^

Health visiting and general practitioners form the net of support for those families who are waiting for assessment or whose children’s needs are not quite high enough to meet the assessment threshold. Thus, as targeted services increase thresholds, health visiting teams are met with greater demands both in volume and in complexity of cases. Our research question is therefore, ‘how are the ‘universal’ and the ‘proportionate’ aspects of proportionate universalist services affected by resource constraint in practice?’

## Methods

### Research synthesis

This paper draws on four studies that examine health visiting in England as a proportionate universalist service as described in table 1 (see also online supplemental material 1).

**Table 1:** Summary of the four studies informing this article

Study title and funding	Study aims	Relevant publications	Ethics approval
*Study 1* **‘**0–5 public health investment in England**’** *Funding:* NIHR (Policy Research Programme), NIHR 203450 *Protocol*^37^	To explore the effectiveness of health visiting in different contexts in England, how it works and at what cost.	Bunting *et al*^29^ Clery *et al*^25^ Woodman *et al*^15^	University College London: REC 20561/002 Health Research Authority and Health and Care Research Wales: REC 23/NE/0138
*Study 2* **‘**Health visiting for families facing adversity**’** *Funding:* NIHR (Public Health Research Programme), NIHR 129901 *Protocol*^38^	To produce a taxonomy describing different patterns of health visiting delivery in England. To assess which health visiting models are most promising for mitigating the impact of maternal adverse childhood experiences. To understand the implications of findings from the DHSC, local authorities and families.	Liu *et al*^28^	University College London, Institute of Education: REC 1531 Health Research Authority and Health and Care Research Wales: REC 24/YH/0167
*Study 3* ‘What do policy colleagues, professionals and families want from the 2½-year review?’ *Funding:* NIHR’s Policy Research Unit through the Child and Family Policy Research Unit (PR-PRU-1217-21301)	To assess recent evidence on tools for measuring child development in children aged 2–2½ years. To understand the views, policy priorities and delivery priorities among key stakeholders for assessing child development at this age. To identify possible alternative tools to the currently used child development tool, based on evidence of reliability, validity, stakeholder priorities and feasibility of implementation in England.	Lysons *et al*^30^ Lysons *et al*^24^	University College London Institute of Education: REC7124
*Study 4* ‘Learning from variation in delivery of the health visiting service in England’ *Funding:* UCL Child Health Research Charitable Incorporated Organisation PhD Studentship, number 563654	To explore the key concepts of disadvantage in child health literature relevant to health visiting. To describe how health visiting contacts vary across England, by local authority and by indicators of disadvantage. To understand why health visiting contacts vary, through the perspectives of health visiting professionals, practitioners and parents and carers.	Clery^31^ Clery *et al*^39^	University College London and Institute of Child Health and Great Ormond Street Hospital Joint R&D Office REC: 20PE25 and 22PE18 University College London REC: 19661/001 NHS Digital REC: DARS-NIC393510-D6H1D Health Research Authority: REC 22/PR/1398

A summary of methods for each study is provided in online supplemental material 1.

DHSC, Department of Health and Social Care; NHS, National Health Service; NIHR, National Institute for Health and Care Research; REC, Research Ethics Committee; UCL, University College London.

Three studies used a mixed-methods design, integrating analyses of routinely collected administrative health visiting data from the Community Services Data Set (CSDS) with thematic analysis of qualitative interviews, while Lysons and colleagues^24^ combined qualitative focus groups with a systematic review. CSDS has a high level of missingness,^25^ so those studies using these data used samples of local authorities that had sufficiently complete data for the period. The mixed-method approach allowed for multifaceted and nuanced descriptions of the service, which this paper now integrates by drawing on the threads of analysis^26^ to explore proportionate universalism in practice. At the outset of this project, the co-authors had been working on separate health visiting studies (table 1) and discussing proportionate universalism, as this was an interesting area of overlap in our work.

Inspired by Critical Interpretive Synthesis (CIS),^27^ we synthesised our existing findings to address a bigger theoretical question, which was initially formulated as, ‘What can we learn about proportionate universalism in practice, using health visiting as a case study?’ In our initial discussions, we observed that most children continued to receive health visiting contacts during a period of decreasing funding and that the service may be finding other ways to manage the resource constraint. As our synthesis came together, we therefore revised our focus as follows: ‘how are the ‘universal’ and the ‘proportionate’ aspects of proportionate universalist services affected by resource constraint in practice?’ This question emerged through iterative discussions that refined both what our data could collectively speak to and what we found to be the core findings through the CIS process (see online supplemental material 2).

The studies share a methodological approach grounded in integrative mixed-methods,^26^ and this shared epistemology facilitated the interpretive work needed for the synthesis. Following CIS, we drew on a purposeful subsample of the literature selected for relevance, aiming to interpret rather than aggregate findings through a synthesis ‘involving both induction and interpretation’.^27^

The findings reported here refer to the 2023 specification of the Healthy Child Programme, with some reflection on the 2026 updates included in the Discussion.

## Findings

### Maintaining reach

Universal health visiting contacts are shaped by how they are measured: a local authority’s ability to deliver all five universal contacts for all eligible families within the time specified for each review (henceforth, its ‘reach’) is a major Key Performance Indicator (KPI) for health visiting under the 2023 specification of the Healthy Child Programme. This is consistent with proportionate universalism’s goal to offer everyone something.^8^ Our studies (1 and 2) found that the reach of the three contacts delivered in the first year of life is generally high: between 2018 and 2020, in 57 local authorities, almost all (89%–99%) eligible children received their new birth contact, 6–8-week review and 1-year review.^28^ Similarly, our longitudinal analysis of 2016–2020 data from 10 local authorities showed that 99% of children had at least one universal contact with the health visiting team by 12 months, and 79% had all three.^29^ While the data regarding the 2–2½-year review showed substantial variation across local authorities, the median reach was 81% (range 33%–98%) of children receiving this final universal contact.^28^

While maintaining a variable but generally high reach of universal contacts, our studies show that most services deliver more additional contacts than universal contacts: administrative data from 57 local authorities showed that, on average, there were 1.6 additional contacts delivered for every universal contact for infants in 2018–2020.^15^ Although this varied between local areas, we found that there were more additional contacts than universal contacts in 80% of these local authorities during this period.^28^ Across 10 local authorities between 2016 and 2020, 63% of children received at least one additional contact in their first year of life, 25% received three or more and 4% received 10 or more before they turned one.^29^ We infer from this that while universal reach is maintained and incentivised, there are drivers that keep the provision of additional contact high, even when resources are diminishing. Interviewees reported delivering additional contacts as part of their professional integrity, despite service pressures:

“yeah, [I] can refer it but, you know, perinatal mental health service have criteria so you have to be pretty bad mental health-wise… and this is where it’s difficult, because as a nurse and with my registration sometimes I will say, ‘well I’m not going to leave this mum and I’ll ring her and make sure everything is okay’ but the service doesn’t encourage me to do that (Health Visitor, Study 1^15^, p.47).

### Balancing needs assessment and health promotion

Our studies suggest the purpose of universal contacts is understood to be twofold. First, health visiting seeks and assesses need and as described in policy, ‘determine(s) targeted interventions which can be met within the services or the need for more specialist interventions that require referrals or clear signposting’ when needs are found.^14^ Parent interviews show that this holistic approach is what they want:

“I would expect just a warm conversation about how your family-, […] and, kind of, having a conversation on what’s going on with you, maybe” (Parent, Study 4^30^).

This is reflected in the way health visitors report valuing universal contacts most for whole-family need elicitation work and how this can be complex:

“…You are there to do a full family strengths and needs assessment, get hold of a family demographic and a configuration so that you can pile together what this looks like, who is this family, who’s staying in the house, who lives here, you know, who’s that bloke that just walked in? Oh, is that your partner? No. Well, who is it then? Oh, we’ve got a lodger. What! Okay, so the lodger is having direct contact with your new baby and the two children that you’ve got in your home, who is this lodger? and things just start to spiral. …and dad’s got four children elsewhere… does he see them, no, he doesn’t have any contact, okay, so who’s determined he doesn’t have any contact then, that’s a bit of a red flag, … hold on, I need to come back, there’s something not quite right here, I need to see her on her own and then you might get the opportunity to talk to her about domestic abuse because and if she’s safe at home, and then she makes a disclosure…” (Health Visitor, Study 1^15^, pg. 36)

Second, health visiting delivers health promotion via universal contacts.^13^ Across studies (Studies 1, 3 and 4), we found that health visitors considered eliciting and assessing needs to be the more important component and consistently reported feeling torn between ‘ticking boxes’ for health promotion and being able to conduct a proper needs assessment:

“A new birth [universal contact] particularly, you’ve got so many things… all your tick-boxing stuff to try and get through. So, it’s that decision of, do I tick all my boxes in this visit today and then come back and see you to deal with the elephant in the room, or do we do the elephant today and I’ll come back and tick boxes another day?” (Health visitor; Study 3^31^, p.267).

### Differentiating universal contacts

While the reach of universal visits is usually over 50%, we found variation in how universal contacts are delivered.^28^ Data from 2018 to 2020 showed that the first two universal contacts predominantly took place in the home, but there was substantial variation in the location of the final two universal contacts.^28^ This is important because analysis of focus group data suggests the location of a universal contact can shape the content: both parents and health visitors report preferring universal contacts in the child’s home, reasoning that this is where the child is most comfortable, where caregivers more easily disclose sensitive issues and where health visiting teams can directly observe home conditions.^30^ As such, families and professionals perceive home contacts as better for needs assessment and elicitation. Where professionals reported a preference for clinic appointments, they expressed the benefit as time efficiency rather than improved quality.^30^

The style of face-to-face contacts can also vary by the number of families being reviewed at once. Our focus group findings showed that some local authorities offer ‘group’ contacts for later reviews, enabling concurrent reviews of multiple children. Seeing a family in a group setting was thought to narrow the focus of what should be a holistic assessment of the whole family^13^ to a specific focus on the child’s development and to be less effective for needs assessment:

“I think [group reviews] are focused on the child and not the family. Because you can’t explore lots of things like maternal mood and her relationships and those sorts of things in a group setting… it’s just not appropriate.” (Health Visitor; Study 3^30^).

Our findings show that the staffing of universal contacts also varies^31^: health visitors usually deliver new birth contacts, but skill-mix colleagues (box 1) often deliver later universal reviews for families with no known needs (Study 1):

“Children [receive] the antenatal which sometimes can just be a phone call, then their new birth and their six to eight week… and then they won’t have any access with the service until the nine month review which will be one of us, as a Band 4 or a Band 3, and then it can be a completely different person at their two year review” (Skill-mix colleague, Study 1,^15^ p.45).

Skill-mix team members have different job descriptions than health visitors. One health visitor described the difference (Study 1):

“[When] I was a Band 5, it didn’t concern me. I was going in to do the job and ticking the boxes and completing the KPIs for families that were low risk. I didn’t even have any professional curiosity, so I’d go in and they’d answer questions and I wouldn’t push further […] as a Band 5 you’re very much walking across the top of it […] because that’s not your remit really.” (Health Visitor,^15^ p.37).

Other health visitors in Study 1 also noted how this approach risked missing needs in families as it was not in the skill-mix remit to ‘explore’, ‘push further’ or ‘uncover’ family needs, but instead to focus on child need^15^ (Study 1, p.45). Based on these data, we abduce that this approach means families with no known needs are less exposed to health visitors’ expertise in building relationships and eliciting family need holistically.

Our synthesis therefore suggests that the delivery of universal contacts to ‘low risk’ families by skill-mix team members results in a differentiation of access to need elicitation within the universal service, in contrast to the universalist principle that ‘everyone needs something’ which underpins proportionate universalism. By filtering families with no known needs towards pathways that are less sensitive to need, the opportunity for their needs to be elicited is reduced, while maintaining high reach. Similarly, our interviews (Studies 1, 3 and 4) suggest that families with complex known needs are more likely to see a fully qualified health visitor than a skill-mix colleague^15 30^ (see below). Interviews with service leads and commissioners (Study 1) suggest that the intention is to reduce the cost of universal contacts and address workforce shortages of fully qualified health visitors, and our synthesis interprets the impact of this approach as being the reduction of the likelihood of identifying new needs. In practice, then, we argue that this approach likely manages or limits the identification of new ‘demand’ for the service.

### Responding to complex needs

The Office for Health Improvement and Disparities’ 2023 guidance suggested how additional contacts may address common needs: ‘for example, at 3 months with support for the continuation of breastfeeding or advice on safer sleep’ and directs health visitors to refer specialist needs to relevant services.^14^ However, interviews with health visitors told us more about how they used additional contacts to provide intensive support to families with multiple and complex needs and less about the sorts of contacts suggested by the 2023 guidance. This may be because additional contacts for low-level needs may be less memorable or feel less significant in an interview context, but some interviewees attributed their accounts of intensive work to the shortage of health visitors and the way that skill-mix colleagues draw the ‘universal’ work out of health visitors’ workload:

“I don’t know whether it’s just that, because there’s less of us, we’re doing more of that kind of [intensive] work… and because we’ve not been able to recruit health visitors which leaves the vulnerable and the more complex families for us to do… as health visitors, we’re expected to have that wide range of knowledge base, all of that training, so that if we’ve got someone on their knees and their mental health has gotten to that point, we know what to do with them and we would manage them under our caseload. If a [skill-mix colleague] was to come across a mum that was really quite poorly with her mental health, they’d be expected to raise that, because their role is universal” (Health Visitor, Study 1^15^ p.42).

Health visiting professionals have described providing intensive support as stressful^15^ (Study 1, p.48), but in some cases parents also described it as positive: “without (the health visitor), things could have gone so much worse” (Parent, Study 1,^15^ p.57), or transformational:

“I’m really grateful to my health visitor… She got the crisis team to come in every day for, like, a while and then… she got me with the perinatal team, and then they took over from the crisis team and were coming every day, and then she was coming every day as well. So, she would like turn up and just like, check on me and the baby and just make sure we’re okay… without her, things could have gone so much worse, like, she kind of stepped in and caught things before they got to the point where I might have lost my baby or I might not have been here, so, yeah. It’s had an impact on me and my life but it’s had a bigger impact, I think, on my children, in that now I am, like, a much better mum than I would have been. So I probably would have thought it was okay to kind of smack them or throw things at them or drag them or threaten them. I probably would have thought it was okay as well to kind of leave them, to go out the house and just leave them alone, because all these things were like what I experienced, and I didn’t understand that that wasn’t okay and that wasn’t normal” (Parent, Study 1^15^ p.42).

This indicates that while intensive work may be affecting delivery of the overall service, in some cases it is supporting meaningful change.

Our synthesis also showed that where families have known complex needs but do not meet thresholds for other services, or are on waiting lists, health visiting teams continue to contact families with needs that would otherwise fall outside their remit of care:

I’ve referred to them as urgent, we went round to speak to her for twelve weeks, twelve weeks. So it’s almost like, ‘if you are not killing yourself right now, we can’t help you, sorry.’ And that’s the biggest thing for me at the minute is our mental health services, it’s shocking, absolutely shocking. And it’s kind of like, we are almost plugging that gap. I’m not a mental health specialist. Yes, I can come and I can sit and I can listen but yes, it’s awful, it’s awful, it really is (Health Visitor, Study 1^15^ p.46).

We interpret these findings across studies to mean that health visiting teams use some additional contacts to ‘hold’ families who have identified needs that health visiting cannot meet, but which health visiting can also not ignore or refer on. These are beyond the needs identified by the 2023 policy, but, as shown in the above quotation, are below thresholds for referral. Proportionate universalism directs that treatment increases in line with need, as is happening here, but in this and other similar examples, the increased treatment is not expected to ameliorate the problem. There is no cap on the number of health visiting contacts a family can have, so this ‘holding’ can be indefinite until the child ages out of the service (Studies 1 and 3). The finding that health visiting uses additional contacts to ‘hold’ high-but-below-referral-threshold needs emerged in all four studies, and from our synthesis, we interpret the wider effect of this, which we abduce, is that the ‘universal’ aspect of the service is being adapted to cope with the demands of the ‘additional’.

As a proportionate universalist service in a landscape of overstretched targeted services, health visiting becomes an open-access holding ground for identified but unmet needs, much of it very complex. At worst, health visiting’s capacity to ‘keep an eye’ on a family without the scope to fully intervene risks becoming a bureaucratic fig leaf covering the absence of services for a family that is not quite meeting the very high threshold for specialist services such as perinatal mental health or children’s social care.

These findings indicate that the intensive work health visitors do with families with known complex needs pulls them towards longer-term supportive work, which is not indicated by the 2023 policy guidance. We argue that this creates an informal threshold for need elicitation that directs families with already known needs towards health visitors, who are highly trained in needs elicitation, and directs families with no known needs to skill-mix colleagues for support and needs assessments, advising them to ask for help if needed.

## Discussion

This study has demonstrated that resource constraint affects the implementation of proportionate universalist services in particular ways. The study has limitations: our focus on a targeted body of research on one service limits the reach of our argument, and our findings on proportionate universalism may hold less true of other proportionate universalist services. Proportionate universalist services which have a clearer and narrower focus, such as cancer screening programmes, may not experience the same issues. We welcome future research to interrogate our conclusions by drawing on a wider spectrum of services and studies. We also highlight the incompleteness of the CSDS, which limits the quantitative analysis of three of the studies included,^25^ and note that fuller quantitative data may have yielded different findings within the synthesis. This limitation was managed within each study by use of stakeholder consultation, namely, sense-checking the data with local authorities to make sure it matched with local records. This means that the data included has been checked for reliability, but the national picture is still unclear. If the CSDS grows more complete in future, then future analyses may challenge or affirm the findings included within this synthesis. Finally, we draw on an integrative mixed-methods approach to ‘follow the thread’^26^ of findings across different types of data: for positivist or strong constructivist approaches, this can be critiqued as either insufficiently reproducible or inappropriately reliant on ‘facts’. Integrative mixed-methods are a pragmatic approach to finding common ground to understand a social issue as part of a multifaceted and multiple approach, and this foundation allowed for the use of CIS to bring four distinct studies together coherently, without overclaiming about the ‘truth’ of the social reality within which this was constructed.

With these limitations in mind, the results from our critical integrative synthesis point to two unresolved issues in the principles of proportionate universalism when put into practice: first, proportionate universalism will always be constrained by resource limitations because it strives to always improve health and reduce inequalities across the social spectrum; and second, the use of thresholds will therefore emerge as a necessary response to resource limitations as services try to align literally finite resources with theoretically infinite need.

The first issue emerged from our synthesis across studies, which showed how different health visiting services have tried to meet rising needs with diminishing financial resources and struggles to recruit trained health visitors, and how this has resulted in a bifurcation of the universal offer. From this, we draw the wider observation that any service that has as wide a remit as health visiting will logically never have enough resources, as there will always be needs to uncover and inequalities to address. In the 2023 Healthy Child Programme, no clear provision was made for how to triage need against limited staffing and resources, and local authorities responded generally by ensuring KPIs, which only counted timely universal contacts, were met. In this sense, resource limitation has impacted the quality of universal health visiting more than it has affected the (measured) ‘quantity’ of health visiting activity. A differentiation of what the ‘universal’ contact looks like for families has emerged, based on whether they already have needs known to the service. Our analysis thus implies that a ‘targeted’ logic, prioritising those with most need, has started to shape the practice of this proportionate universalist service, as health visiting’s most skilled staff work with those families with already known needs, particularly if those needs are complex. However, health visitors report that their defining skillset is *needs elicitation*, and this is often not the core need for families with known needs: they are already seeking support. By contrast, health visitors frame universal contacts for those families with no known needs as a crucial opportunity for need assessment and elicitation. However, within the differentiated model of universal contacts, this opportunity is currently being widely met by colleagues who are reportedly less skilled in need elicitation. Across all studies, we heard that leaders and practitioners know that this is a problem and that this situation has arisen not by design, but through attempts to meet service demands with limited resources.

The policy implications of this are twofold (box 2). First, the recent NHS 10-year plan targets improved quality, as well as high reach, for health visiting.^3^ We argue that the quality of health visiting is diminished when health visitors are pulled away from needs elicitation in a proportionate *universalist* service and towards supporting families’ complex needs in a *proportionate* universalist service, filling in where specialist services are overwhelmed. This is a core dilemma for this particular service’s identity and must be addressed if health visiting’s quality is to improve in a sustained, coherent way: without effective needs elicitation, health visiting will either become a de facto universal-only service offering everyone much the same regardless of their circumstances or a de facto targeted-only service, which attends primarily to those already flagged as having additional needs. The 2026 Healthy Child Programme’s introduction of reporting on the practitioner undertaking a contact, the place of the contact and any additional targeted health and development reviews will all likely shape how health visiting resources universal contacts in future and may change the emerging pattern of differentiated universal contacts, if there are sufficient health visitors to staff all universal contacts, as the new guidance directs.

Box 2 **Learning points for proportionate universalist services working under resource constraint**

- To avoid becoming a de facto targeted-only service, the relationship between the universal service and the proportional, additional work must be clearly defined within the service design: the core function and quality of the universal dimension must be protected, even when demands on the proportional end are high, if it is to remain meaningfully universal.
- To avoid becoming a de facto universal-only service, the scope of proportional work must be clearly defined and agreed on, with coherent service-wide guidance given on steps to take when referral services are absent or operating under heightened thresholds. If a proportionate universalist service is the only service offering support to a client who has needs that are higher than the service can support, but below referrable thresholds, then there is an obvious moral hazard and health risk in limiting or cutting off that support in order to protect the universal service. At the same time, it must be recognised that the referring service is likely not the best placed to support these referrable needs, and this tension impacts the delivery of the service as a whole.
- The Key Performance Indicators for proportionate universalist services must take into consideration both the universal and proportionate elements of the service to coherently reflect and incentivise the proper working of the relationship between these two components: this will be individual to each type of proportionate universalist service.

More widely, we argue that our synthesis provides policy and practice lessons for other proportionate universalist services: in any such service, needs elicitation must be conceptualised as a priority for the universal element, because this is the mechanism by which further need can first be identified and then addressed. Further, if a proportionate universalist service must rely on referring to targeted services as part of its operating logic, then the service must have clear operational boundaries, and the targeted services’ thresholds must be resourced sufficiently to adjoin those boundaries to avoid ‘gaps’ between services. Without this safeguard, our analyses suggest that proportionate universalist services end up ‘holding’ cases waiting for referral and risk the moral hazard of identifying needs that cannot be met.

The second issue our synthesis highlights is theoretical: we call for further development of the principles of proportionate universalism to address the challenge of de facto thresholding within proportionate universalist services as a response to resource constraint; and a clearer articulation of how improving health universally while reducing health inequalities across the spectrum can be squared with limited resources overall. Proportionate universalism has the attractive goal of both improving health across the social gradient and reducing that gradient: it is important to find ways to implement this approach, which honours the principle of equity in valuing all lives. To do this, we must think practically and innovatively about how to prevent resource constraint from selling us short of the prize.

## Supplementary material

10.1136/jech-2026-225934 online supplemental file 1

## Data Availability

No data are available.
