# Supplementary material for: How are proportionate universalist services affected by resource constraint in practice? Exploring implementation challenges using a synthesis of four health visiting studies in England
Source: J Epidemiol Community Health. 2026 Jul 16;80(11):e225934. doi: 10.1136/jech-2026-225934 (PMC13691698; doi:10.1136/jech-2026-225934)
Supplement: Supplementary file 1 [file jech-80-11-s001.pdf]

Supplementary Material 1: Summary of methods and findings from four health visiting studies

Study title and funding	Aims	Quantitative sample and methods	Qualitative sample and methods	Findings	Ethics Approval
<u>**Study 1**</u><br><br>**0-5 Public Health Investment in England**<br><br>*Funding:* National Institute for Health and Care Research Policy Research Programme, NIHR 203450<br><br>*Protocol*: Harron *et al*., 2023.<br><br>*Publications*: Bunting *et al.*, 2024; Bunting *et al.*, 2025; Clery *et al.,* 2024; Woodman *et al.,* 2025	(1) To explore the effectiveness of health visiting in different contexts in England, how it works, and at what cost.	<u>Objective</u>: to describe health visiting activity in the first year of a child's life.<br><br>A longitudinal analysis of universal and additional recorded health visiting contacts in first year of life for 52, 555 children born in NHS hospitals in the UK between April 2016 and March 2020, across 10 local authorities which had complete data for five consecutive quarters (15 months) recorded in CSDS.	<u>Objective</u>: to explain and contextualise the results from the quantitative analysis; to provide an in-depth description and programme theory of health visiting practices and variations<br><br>53 interviews across three local authorities with health visiting team members, including skill-mix (n=30) and parents (n=23); and approximately 120 hours of observation of health visiting practice, conducted between August 2023-August 2024. Analysed using thematic analysis.	<u>Quantitative</u>:<br>There is high reach of health visiting in first year of life; 99% of children receive at least one contact in their first year of life. Additional contacts are also widespread, with 63% of children receiving one or more contacts in the first year of life. There are high levels of variation in reach of contacts by local area, as well as by duration and type of contact, and some social patterning.<br><br><u>Qualitative</u>:<br>Different ways of understanding health visiting's ways of working were identified, with particularly identifying tensions between how good health visiting is imagined by parents and health visitors, and what the work actually done entails in a context of resource scarcity. KPIs do not capture the most impactful or intensive work health visitors do through additional contacts.	University College London, REC 20561/002<br><br>Health Research Authority and Health and Care Research Wales REC 23/NE/0138
<u>**Study 2**</u><br><br>**Health Visiting for Families Facing Adversity**<br><br>*Funding:* National Institute for Health and Care Public Health Research Programme NIHR 129901<br><br>*Protocol:* Woodman *et al.,* 2022.<br><br>*Publications:* Liu *et al.,* 2024	(1) To produce a taxonomy describing different patterns of health visiting delivery in England;<br>(2) to assess which health visiting models are most promising for mitigating the impact of maternal Adverse Childhood Experiences<br>(3) to understand the implications of findings from the Department of Health and Social Care, local authorities and families	<u>Objective</u>: to describe the reach of health visiting for families with markers of adversity.<br><br>A cross-sectional analysis of health visiting contacts in 57 local authorities which had complete data on universal health visiting contacts for at least one quarter (3 months) in CSDS between April 2018 and March 2020.	*The qualitative analyses for this study are on-going and are therefore excluded from this paper.*	<u>Quantitative</u>:<br>Almost all eligible children received the universal contacts within the first year of life (89 – 90%) and 80% of the included local authorities delivered more additional than universal contacts. Duration of contacts varied significantly, as did the percentage of contacts delivered in the home.	University College London, Institute of Education: REC1531;<br><br>Health Research Authority and Health and Care Research Wales REC 24/YH/0167

<u>**Study 3**</u><br>**What do policy colleagues, professionals and families want from the 2 ½ year review?**<br><br>*Funding:* National Institute for Health and Care Research's Policy Research Unit through the Child and Family Policy Research UnitPR-PRU-1217-21301.<br><br>*Publications:* Lysons *et al.*, 2024;<br>Lysons *et al.*, 2025	(1) To assess recent evidence for tools measuring child development for children aged 2-2½ years;<br>(2) to understand the views and policy and delivery priorities among key stakeholders for assessing child development at this age;<br>(3)to identify possible alternative tools to the currently used child development tool, based on evidence of reliability, validity, stakeholder priorities and feasibility of implementation in England.	*<u>Objective</u>: to conduct a systematic review of available child development tools which could be used for the 2 – 2 ½ year review.*<br><br>*The systematic review findings are excluded as they are not relevant to this paper.*	<u>Objective</u>: to examine stakeholder views and experiences related to assessing child development at the 2–2½-year health and development review in England, including priorities for the universal tool used to measure child development.<br><br>15 focus groups with 29 parents, 24 health visiting team members, 5 service managers and five policy colleagues (January – September 2023), analysed using reflexive thematic analysis.	<u>Qualitative</u>:<br>Two themes were constructed: that the ASQ-3 is "just part of the puzzle: a useful tool in a holistic review," which enables wider conversations in a whole-family (not just child-centred) approach; second, "they need to know why they're doing it: a need for clarification" highlights stakeholder views that the use of child development tools in the final universal review needs to be delivered with a clear understanding of its intended purpose, which is to provide population-level data.	University College London Institute of Education: REC7124
<u>**Study 4**</u><br><br>**Learning from variation in delivery of the health visiting service in England**<br><br>*Funding:* UCL Child Health Research Charitable Incorporated Organisation PhD Studentship, number: 563654<br><br>*Publications:* Clery, 2023;<br>Clery *et al.,* 2025.	(1) To explore the key concepts of disadvantage in child health literature relevant to health visiting;<br>(2) to describe how health visiting contacts vary across England, by local authority and by indicators of disadvantage;<br>(3) to understand why health visiting contacts vary, through the perspectives of health visiting professionals, practitioners, and parents and carers.	<u>Objective</u>: to describe variation in health visiting in different areas.<br><br>CSDS data recording 3,566,840 children under 5 known to health visiting services in the 2 financial years from April 2018 until March 2020; local health visiting records as held by two local authorities for 2016- 2022; and records of contacts held by the Family Nurse Partnership from 2010 - 2020 as an example of a targeted health visiting programme.	<u>Objectives</u>: to understand how different groups perceive and experience the role of and support offered by the health visiting service in their local area; and to explore the reasons why the health visiting service varies within and between LAs.<br><br>14 interviews with health visiting team members (n=9), parents (n=4) and one commissioner across three purposefully sampled local authorities, collected in 2023. Analysed using thematic analysis.	<u>Quantitative</u>:<br>Delivery of health visiting is highly variable between areas in England. The patterning of delivery of universal contacts according to indicators of need differs compared to the patterning for additional contacts: children living in the most deprived neighbourhoods as measured by Index of Multiple of Deprivation were less likely to receive their universal contacts, but more likely to receive additional contacts; and more likely than children in less deprived areas to receive their 2 – 2 ½ year review.<br><br><u>Qualitative</u>:<br>Factors beyond just local area need contribute to variation in service delivery, including availability of community and specialist services; rigidity of KPI focus, impact of commissioning models and service changes, and staff values.	University College London, Institute of Child Health and Great Ormond Street Hospital Joint R&D Office, REC: 20PE25<br><br>University College London REC: 19661/001<br><br>NHS Digital REC: DARS-NIC393510-D6H1D<br><br>University College London Institute of Child Health and Great Ormond Street Hospital Joint R&D Office: ref number: 22PE18<br><br>Health Research Authority: ref number: 22/PR/1398

Supplementary Material 2: Details of the Critical Interpretive Synthesis process:

Our synthesis meetings were attended by the study members most involved in the data analysis in each study (all represented here as co-authors), so we also benefitted from the deep understanding of the data which the original data collectors and analysts held, as well from the published outcomes. While CIS was originally envisioned for new concept or theory creation from data [32] we aim to use empirical findings to explore an existing theory, rather than building a new concept. Nevertheless, this means we focus "on the development of concepts and theory rather than on exhaustive summary of all data" [32], hence our small, focussed sample.

The synthesis meetings were led by JW, who had a leading or supervisory role in all four studies, and who initiated the interest in looking at proportionate universalism through the lens of this body of work. She brought the following questions to the first synthesis meeting on the basis of her own close knowledge and understanding of the studies:

- What does "universal" look like in practice?
- What does "more" look like in practice?
- What is the balance of proportionate to universal work?
- How do families get filtered into receiving "more"?

Co-authors contributed insights from their own study findings, and through discussion, negotiated the key points and issues from empirical data to answer these theoretically-driven questions. JW drafted the first synthesis which was then circulated to the co-authors with some points being agreed to be folded together or removed as 'additional' to the core argument, and others expanded to be better contextualised with findings.

We presented our initial synthesis at the International Children and Families Conference in Bristol, UK in June 2025, to test the emerging findings and seek feedback from an expert audience. The feedback confirmed the logic of our approach and the coherence of the synthesis, so did not prompt major revisions but did help us develop a clearer articulation of structure and argumentation. Subsequently, AL led the drafting of the article based on the feedback and initial synthesis. This involved close reading of notes of the discussion meetings and publications of the original studies, the circulation of drafts in three rounds to all co-authors for comment, and a final meeting to agree the final output. This allowed the data analysis leads to scrutinise the synthesis to ensure fidelity with their original study findings.

The studies included in the synthesis were all conducted under the 2023 iteration of the Healthy Child Programme, which was updated in 2026 while this article was under review. The refreshed version includes updated Key Performance Indicators, and more detailed descriptions of the kind of work health visiting services are expected to do with families experiencing specialist support for safeguarding or special educational needs and disabilities.
